# Vitamin D receptor gene BsmI, FokI, ApaI and TaqI polymorphisms and the risk of systemic lupus erythematosus

**DOI:** 10.1007/s11033-012-2118-6

**Published:** 2012-10-14

**Authors:** Adrianna Mostowska, Margarita Lianeri, Mariusz Wudarski, Marzena Olesińska, Paweł P. Jagodziński

**Affiliations:** 1 Department of Biochemistry and Molecular Biology, Poznań University of Medical Sciences, 6 Święcickiego St, 60-781 Poznan, Poland; 2 Institute of Rheumatology, Warsaw, Poland

**Keywords:** VDR polymorphism, SLE, PCR–RFLP

## Abstract

Recently, several studies have demonstrated the role of vitamin D receptor (*VDR*) polymorphisms in the development of systemic lupus erythematosus (SLE); however, these results are inconsistent between different cohorts. Therefore, we studied the prevalence of the *VDR* FokI (rs2228570), BsmI (rs1544410), ApaI (rs7975232) and TaqI (rs731236) genotypes and alleles in SLE patients (*n* = 258) and healthy individuals (*n* = 545) in a Polish population. We did not observe significant differences for either the *VDR* FokI, BsmI, ApaI and TaqI genotype and allele frequencies in patients with SLE and healthy individuals. However, the frequency of the *VDR* F/F and F/f genotypes of FokI was statistically different between patients with renal disease and patients without this symptom OR = 3.228 (1.534–6.792, *p* = 0.0014), *p*_corr_ = 0.0476)]. There was no association of the studied *VDR* BsmI, ApaI and TaqI polymorphisms with clinical manifestations and laboratory profiles in patients with SLE. Our study indicates that the studied *VDR* FokI variant might increase the risk of some clinical presentations in patients with SLE.

**Electronic supplementary material:**

The online version of this article (doi:10.1007/s11033-012-2118-6) contains supplementary material, which is available to authorized users.

## Introduction

Systemic lupus erythematosus (SLE) is an autoimmune disease for which the underlying cause remains unclear [1]. The putative causations include interactions between environmental factors, disease-prone genetic background, and various pathogen eliciting innate and adaptive immune responses [2–4]. Hyperactivation of the immune system results in the overproduction of autoantibodies and in the formation of immune complexes [1]. These immune complexes are deposited in various organs and tissues, causing the symptoms seen in the clinical manifestations of SLE [1].

Some studies have suggested the possible role of vitamin D in the development of rheumatoid arthritis (RA), type 1 diabetes (T1D), Crohn’s disease, multiple sclerosis (MS) and SLE [5–7]. The primary function of the active form of vitamin D, 1,25-dihydroxyvitamin D3 [1,25(OH)_2_D_3_], is calcium homeostasis and bone metabolism [7, 8]. In recent years, the function of vitamin D has been further studied and determined to include this molecule as a pleiotropic regulator of human physiology, as having a role in cancer chemoprevention, and as playing a role in cardio-protection and immune system modulation [7, 8].

Vitamin D transmits signals to target cells using the vitamin D receptor (VDR), which is composed of both ligand binding and conserved DNA binding domains. The conserved DNA binding domains function primarily as regulators of gene transcription [9]. VDR forms heterodimers with the related retinoid X receptors and binds to DNA to initiate histone modifications, chromatin rebuilding, and RNA polymerase II binding, which is essential for initiation of transcription [10]. The *VDR* gene is located on chromosome 12q and contains more than 470 single nucleotide polymorphisms (SNPs), some of which modulate 1,25(OH)_2_D_3_ uptake. Therefore, these SNPs can lead to this gene’s polymorphisms to being considered as candidate disease risk variants [11]. The most commonly studied *VDR* SNPs include rs10735810/rs2228570 (FokI) situated in exon 2, and three SNPs in linkage disequilibrium, namely rs1544410 (BsmI) located in intron 8, rs731236 (TaqI), and rs7975232 (ApaI), the last being an SNP situated in exon 9 and intron 9 [8, 12].

Recently, several studies have demonstrated the role of *VDR* SNPs in the development of SLE and its clinical manifestations; however, these results are inconsistent between different cohorts [13–19]. Therefore, we aimed to study whether the FokI, BsmI, ApaI and TaqI SNPs can be a genetic risk factor of SLE in the Polish population. Because SLE is a heterogeneous disease, we also evaluated the association of these SNPs with different SLE symptoms.

## Patients and methods

### Patients and controls

Two hundred and fifty-eight patients (women only) fulfilling the American College of Rheumatology Classification criteria for SLE [20, 21] were selected randomly at Institute of Rheumatology in Warsaw, Poland (Table 1). The five hundred and forty-five controls consisted of randomly selected unrelated healthy female blood donors and healthy women who had an examination at the Institute of Mother and Child in Warsaw, Poland. Both patients and control groups were of Polish Caucasian origin. The protocol of the study was approved by the Local Ethical Committee of Poznań University of Medical Sciences. Written agreement was obtained from all participating individuals. The mean age of SLE patients at diagnosis was 40 ± 11 years, and of controls 39 ± 10 years.

**Table 1:** Clinical manifestations in SLE patients

Characteritic	Numbers of patients
Malar rash	40
Discoid rash	73
Photosensitivity	119
Oral or nasopharyngeal	103
Arthritis	36
Serositis	28
Renal	131
Neurologic	54
Hematologic	88
Immunologic	120
Antinuclear antibody	258

### Genotyping

DNA was isolated from peripheral leucocytes using a standard salting out procedure. The presence of the *VDR* FokI (rs2228570), BsmI (rs1544410), ApaI (rs7975232) and TaqI (rs731236) SNPs was identified by polymerase chain reaction-restriction fragment length polymorphism (PCR–RFLP) according to the manufacturer’s instructions New England BioLabs (Ipswich, USA). DNA fragments were separated in 3 % agarose gels and visualized by ethidium bromide staining. Primer sequences and conditions for PCR–RFLP analyses are presented in Supplemental Table 1S. The presence of the FokI, BsmI, ApaI and TaqI polymorphisms was also confirmed by repeated PCR–RFLP analysis and commercial sequencing.

### Statistical analysis

The distribution of genotypes in patients and controls was tested for deviation from Hardy–Weinberg equilibrium. Differences in genotypic and allelic distribution between patients and controls and associations between clinical manifestations, production of antibodies (Abs), and polymorphism distribution in patients were determined by Chi-square (χ^2^) or Fisher exact test. Bonferroni correction for multiple comparisons was used and both *p* values, before (*p*) and after correction (*p*_corr_), were determined. The FokI and BsmI polymorphisms were tested for association with SLE using the Chi-square test for trend (*p*_trend_). The odds ratio (OR) and 95 % confidence intervals (95 % CI) were also determined. Statistical significance was interpreted as *p* value <0.05.

## Results

Genotype analysis of the *VDR* FokI, BsmI, ApaI and TaqI polymorphisms did not show a significant deviation form Hardy–Weinberg equilibrium in the SLE and control groups.

### Distribution of the *VDR* FokI (rs2228570) and BsmI (rs1544410) genotypes and alleles in SLE patients and healthy individuals

We observed an increased frequency of the F/F genotype in patients as compared to controls, but these differences were not significant, with OR of the F/F versus f/fgenotypes 1.300 (95 % CI = 0.840–2.010, *p* = 0.238) (Table 2). The frequency of the F/f genotype in patients and controls was similar at 0.44 and 0.45, respectively, and the OR for the F/f versus f/f genotypes was 1.163 (95 % CI = 0.757–1.786, *p* = 0.491) (Table 2). There was an increased frequency of the F/F and F/f genotypes in patients compared to controls, but these differences were also not significant OR = 1.225 (95 % CI = 0.820–1.829, *p* = 0.321) (Table 2).We also observed an increased frequency of the F allele in patients with SLE as compared to the control group; however, these differences were not significant, OR = 1.146 (95 % CI = 0.924–1.421, *p* = 0.215). The *p* value of the Chi-square test for the trend observed for the FokI polymorphism was also not statistically significant (*p*_trend_ = 0.232).

**Table 2:** Distribution of the FokI (rs2228570), BsmI (rs1544410), ApaI (rs7975232) and TaqI (rs731236) SNPs in SLE

rs no.	Genotype	Patients (frequency)	Controls (frequency)	Odds ratio (95 % CI)	*p* ^a^	*p*_trend_
rs2228570	ff	40 (0.15)	100 (0.18)	Referent		
	fF	113 (0.44)	243 (0.45)	1.163 (0.757–1.786)	0.491	
	FF	105 (0.41)	202 (0.37)	1.300 (0.840–2.010)	0.238	0.2315
	fF + FF	218 (0.84)	445 (0.82)	1.225 (0.820–1.829)	0.321	
	F allele	0.63	0.59	1.146 (0.924–1.421)	0.215	
rs1544410	bb	109 (0.42)	218 (0.40)	Referent		
	bB	121 (0.47)	245 (0.45)	0.988 (0.719–1.356)	0.939	
	BB	28 (0.11)	82 (0.15)	0.683 (0.419–1.111)	0.123	0.2711
	bA + BB	149 (0.58)	327 (0.60)	0.911 (0.675–1.231)	0.545	
	B allele	0.34	0.38	0.869 (0.698–1.083)	0.211	
rs7975232	aa	62 (0.24)	136 (0.25)	Referent		
	aA	118 (0.46)	257 (0.47)	1.007 (0.695–1.460)	0.970	
	AA	78 (0.30)	152 (0.28)	1.126 (0.750–1.689)	0.568	0.5533
	aA + AA	196 (0.76)	409 (0.75)	1.051 (0.744–1.485)	0.777	
	A allele	0.53	0.51	1.068 (0.866–1.317)	0.541	
rs731236	tt	28 (0.11)	81 (0.15)	Referent		
	tT	122 (0.47)	247 (0.45)	1.429 (0.883–2.312)	0.145	
	TT	108 (0.42)	217 (0.40)	1.440 (0.884–2.345)	0.142	0.2417
	tT + TT	230 (0.89)	464 (0.85)	1.434 (0.907–2.267)	0.121	
	T allele	0.66	0.62	1.140 (0.916–1.420)	0.240	

^a^Chi-square analysis

There was also no contribution of the *VDR* BsmI polymorphism to SLE. The frequency of the BB genotype was lower in patients with SLE compared to the control group, but not significantly so. OR of the B/B versus b/b genotypes was 0.683 (95 % CI = 0.419–1.111, *p* = 0.123). We found an increase in B/b heterozygote frequency in patients than in controls, but these differences were also not significant, OR of B/b versus b/b genotype was 0.988 (95 % CI = 0.719–1.356, *p* = 0.939) (Table 2). The frequency of the B/B or B/b genotypes was slightly lower in SLE patients, OR of B/B or B/b versus b/b was 0.9113 (95 % CI = 0.675–1.231, *p* = 0.545) (Table 2). We also did not find a significant difference in the prevalence of B alleles between patients and controls. OR for the *VDR*B allele frequency was 0.869 (95 % CI = 0.698–1.083, *p* = 0.211). The *p* value of the Chi-square test for the trend observed for the BsmI polymorphism was also not statistically significant (*p*_trend_ = 0.271).

### Distribution of the *VDR* ApaI (rs7975232) and TaqI (rs731236) genotypes and alleles in SLE patients and healthy individuals

We did not observe an association of the *VDR* ApaI and TaqI SNPs with SLE. OR of the A/A versus a/a genotypes was 1.126 (95 % CI = 0.750–1.689, *p* = 0.568). There was a slight decrease in A/a heterozygote frequency in patients than in controls, OR of A/a versus a/a genotype was 1.007 (95 % CI = 0.695–1.460, *p* = 0.970) (Table 2). The frequency of the A/A or A/a genotypes was slightly increased in SLE patients, OR of A/A or A/a versus a/a was 1.051 (95 % CI = 0.744–1.485, *p* = 0.777) (Table 2). There was no significant difference in the prevalence of A alleles between patients and controls. OR for the *VDR* A allele frequency was 1.068 (95 % CI = 0.866–1.317, *p* = 0.541). The *p* value of the Chi-square test for the trend observed for the ApaI polymorphism was also not statistically significant (*p*_trend_ = 0.5533) (Table 2).

There was also no contribution of the *VDR* TaqI polymorphism to SLE. The frequency of the TT genotype was slightly increased in SLE patients than in the control group. OR of the T/T versus t/t genotype was 1.440 (95 % CI = 0.884–2.345, *p* = 0.142). We also observed a slight increase in T/t heterozygote frequency in patients than in controls, OR of T/t versus t/t genotype was 1.429 (95 % CI = 0.883–2.312, *p* = 0.145) (Table 2). The frequency of the T/T or T/t genotypes was also slightly increased in SLE patients, OR of T/T or T/t versus t/t was 1.434 (95 % CI = 0.907–2.267, *p* = 0.121) (Table 2). We also did not find a significant difference in the prevalence of T alleles between patients and controls. OR for the *VDR* T allele frequency was 1.140 (95 % CI = 0.916–1.420, *p* = 0.240). The *p* value of the Chi-square test for the trend observed for the TaqI polymorphism was also not statistically significant (*p*_trend_ = 0.2417).

### Association of the *VDR* FokI (rs2228570), BsmI (rs1544410), ApaI (rs7975232) and TaqI (rs731236) polymorphisms with clinical symptoms of SLE

**Fig. 1:**
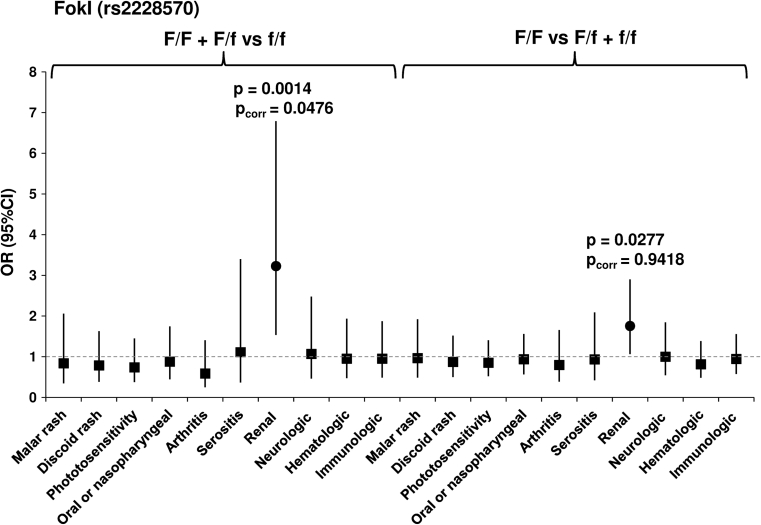
Odds ratio plot for comparison of genotypes *VDR* F/F + F/f versus f/f and F/F versus F/f + f/f between patients with and patients without a particular manifestation. Each OR value is represented by the corresponding *black square* or *circle* with arms representing 95 % confidence intervals (95 % CI). The analysis was performed by χ^2^ test. The *black circle* indicates a significant association

**Fig. 2:**
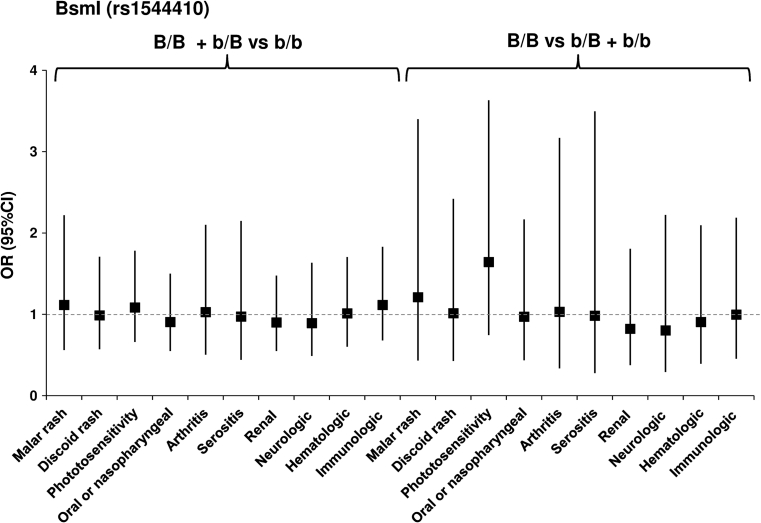
Odds ratio plot for comparison of genotypes *VDR* B/B + b/B versus b/b and B/B versus b/B + b/b between patients with and patients without a particular manifestation. Each OR value is represented by the corresponding *black square* with arms representing 95 % confidence intervals (95 % CI). The analysis was performed by χ^2^ test

**Fig. 3:**
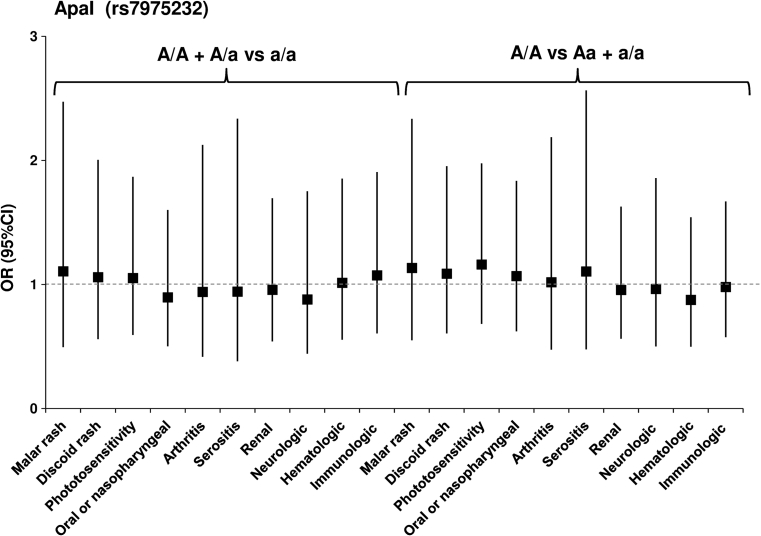
Odds ratio plot for comparison of genotypes *VDR* A/A + A/a versus a/a and A/A versus A/a + a/a between patients with and patients without a particular manifestation. Each OR value is represented by the corresponding *black square* or *circle* with arms representing 95 % confidence intervals (95 % CI). The analysis was performed by χ^2^ test

**Fig. 4:**
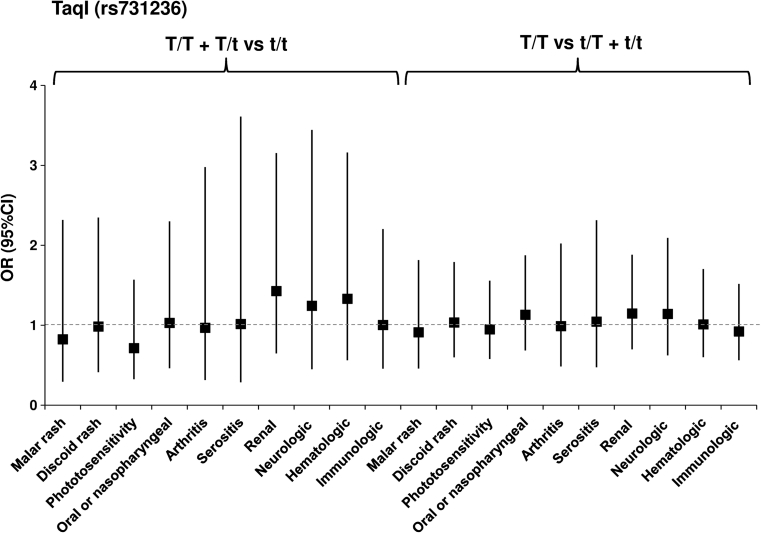
Odds ratio plot for comparison of genotypes *VDR* T/T + T/t versus t/t and T/T versus t/T + t/t between patients with and patients without a particular manifestation. Each OR value is represented by the corresponding *black square* with arms representing 95 % confidence intervals (95 % CI). The analysis was performed by χ^2^ test

Since previous studies indicated the association of *VDR* polymorphisms to some clinical SLE manifestations [13–19], we evaluated their contribution to clinical manifestations present in the patient group we studied. We found a significant association between the F/F and F/f allele with renal manifestations of SLE OR = 3.228 (1.534–6.792, *p* = 0.0014), *p*_corr_ = 0.0476) (Fig. 1; Table 2S, on line supplementary data). However, we did not observe a significant association of the BsmI, ApaI and TaqI polymorphisms with clinical manifestations in patients (Figs. 2, 3, 4; Tables 3S–5S, online supplementary data). There was also no association of either of the studied polymorphisms with the presence of anti-dsDNA, anti–Smith, anti-snRNP, anti-Ro, anti-Scl-70 or anti-phospholipid Abs (not shown).

## Discussion

The immune system in patients with SLE is characterized by an enhanced humoral response and decreased T cell cytotoxicity [22–24]. Immune cells exhibit abnormal signaling, defective gene expression, and changes in cytokine production [22–24]. Vitamin D functions to regulate various immune cells, and an abnormal vitamin D level has been documented in SLE patients in various populations [25–31]. Low vitamin D levels were associated with higher disease activity in Malaysian, Egyptian, Chinese, Israeli and European cohorts [27–31]. The decline of vitamin D concentration in SLE patients was associated with the presence of aortic stiffness, leucopenia, renal disease, increased anti-C1q and anti-dsDNA titers, dyslipidemia and increased cardiovascular risk [29, 30, 32–34]. Recently, Birmingham et al. [35] suggested that a seasonal decrease in vitamin D levels may trigger an SLE flare in non-African Americans. The role of vitamin D in the development of SLE has also been studied in the murine model. Administration of 1,25(OH)_2_D_3_ to mice with SLE resulted in a protective role in the development of this autoimmune disease [36].

Studies performed in vitro have demonstrated that vitamin D inhibits T cell proliferation and leads to a reduced production of interferon-gamma(IFN-γ),interleukin-2 (IL-2), IL-6, IL-12, IL-23 and IL-17 [37–40]. Moreover, the immunosuppressive activity of 1,25(OH)_2_D_3_ is demonstrated by an inhibition in the maturation, differentiation, activation, and survival of dendritic cells, leading to T cell hyporesponsiveness [41]. VDR have been found in monocytes, macrophages, dendritic cells, and effector/memory T cells [25]. This suggests that functional polymorphisms in the *VDR* gene may modulate the immune system and an individual’s susceptibility to developing SLE.

We did not find that the FokI, BsmI, ApaI and TaqI polymorphisms are associated with an individual’s susceptibility to SLE in a Polish population. Our observations are consistent with the findings of Monticielo et al. [18], who demonstrated no association of the BsmI and FokI polymorphisms with the development of SLE in a Brazilian-European cohort. There was also no association of the FokI *VDR* polymorphism with Chinese SLE patients in Taiwan, or of the BsmI *VDR* polymorphism with SLE in Iranian and Thai populations [15–17]. However, the BB genotype of the BsmI *VDR* SNP was found to be a risk factor of SLE in Taiwanese and Japanese populations [13, 14]. In addition to these findings, Luo et al. [19] observed a significantly increased frequency of the B allele in SLE patients from a Han Chinese population.

In our study, SLE patients with the F/F and F/f genotypes of the FokI *VDR* polymorphism exhibited a significantly increased risk of developing renal disease. However, we did not find an association of the BsmI *VDR* polymorphism with clinical manifestations of SLE. There was no association between the BsmI polymorphism and clinical manifestations, laboratory profiles, or lupus nephritisin Chinese SLE patients in Taiwan [14]. However, the *VDR* B allele was associated with the development of nephritis in a Han Chinese population, and the bb genotype was associated with lupus nephritis in a Japanese population [13, 19].

The differences observed between the BsmI and FokI *VDR* polymorphisms and the susceptibility to SLE development and the occurrence of some clinical manifestations may be due to exposure of the analysed groups to different environmental factors, group size and genetic heterogeneity.

The roles of the BsmI and FokI polymorphisms on the function of the VDR receptor have been already determined in several studies [12, 18, 19]. Arai et al. [12] demonstrated that the FokI polymorphism located in exon 2 is linked to a second methionine start site, leading to the formation of a shorter protein receptor that has greater transcriptional activity than the wild type receptor. Recently, Monticielo et al. [18] reinforced the functional role of the FokI polymorphism. They demonstrated that vitamin D concentration was significantly increased in individuals with the f/f genotype versus patients having the F/F genotype [18]. The BsmI polymorphism can be linked to a variable-length polyadenylate sequence within the 3′-untranslated region [8]. Recently, Luo et al. [19] demonstrated that the level of VDR mRNA was significantly decreased in patients with the *VDR* B allele versus those not bearing the B allele.

The BsmI and/or FokI *VDR* polymorphisms have also been recognized as risk factors of some other autoimmune diseases, including RA, Behçet’s, Graves’ and Addison’s diseases, psoriasis, MS, T1D, and others [42–48]. Moreover, both of these polymorphisms have been determined to be risk factors for colorectal, breast, prostate, and other cancers [11].

Our study did not demonstrate that the FokI, BsmI, ApaI and TaqI *VDR* SNPs are risk factors of SLE in the Polish population, but we found an association of the FokI polymorphism with renal manifestations in SLE patients. However, to study the detailed role of these SNPs in SLE, this evaluation should be replicated in other independent cohorts.

## Electronic supplementary material

Below is the link to the electronic supplementary material.

Supplementary material 1 (DOC 117 kb)

Supplementary material 2 (DOC 38 kb)
