# Supplementary material for: Vitamin D receptor gene BsmI, FokI, ApaI and TaqI polymorphisms and the risk of systemic lupus erythematosus
Source: Mol Biol Rep. 2012 Oct 14;40(2):803–10. doi: 10.1007/s11033-012-2118-6 (PMC3538008; doi:10.1007/s11033-012-2118-6)
Supplement: Supplementary file 1 — Supplementary material 1 (DOC 117 kb) [file 11033_2012_2118_MOESM1_ESM.doc]

**Table 2S.** **Prevalence of the FokI (rs2228570) genotypes among SLE patients with different clinical manifestations.**

| **Characteristic** | **Genotype distribution** | | | |  |
| --- | --- | --- | --- | --- | --- |
|  | **F/F**  **(105)^a^** | **F/f**  **(113)^a^** | **f/f**  **(40)^a^** | **Odds ratio (95% CI)^b^, p^c^**  **F/F + F/f vs f/f** | **Odds ratio (95% CI)^d^, p^c^**  **F/F vs Ff + f/f** |
| Malar rash | 16 | 17 | 7 | 0.841 (0.343 - 2.060), 0.704 | 0.966 (0.486 - 1.923), 0.922 |
| Discoid rash | 28 | 32 | 13 | 0.789 (0.382 - 1.629), 0.521 | 0.873 (0.501 - 1.520), 0.631 |
| Phototosensitivity | 46 | 52 | 21 | 0.739 (0.376 - 1.452), 0.379 | 0.854 (0.519 - 1.408), 0.537 |
| Oral or nasopharyngeal | 41 | 45 | 17 | 0.882 (0.445 - 1.746), 0.717 | 0.940 (0.566 - 1.563), 0.812 |
| Arthritis | 13 | 15 | 8 | 0.590 (0.247 - 1.408), 0.230 | 0.799 (0.385 - 1.659), 0.546 |
| Serositis | 11 | 13 | 4 | 1.113 (0.364 - 3.402), 1.000^e^ | 0.936 (0.419 - 2.090), 0.872 |
| Renal | 62 | 58 | 11 | **3.228 (1.534 - 6.792), 0.001** | **1.755 (1.062 - 2.902), 0.028** |
| Neurologic | 22 | 24 | 8 | 1.070 (0.462 - 2.479), 0.875 | 1.002 (0.544 - 1.846), 0.994 |
| Hematologic | 33 | 41 | 14 | 0.954 (0.470 - 1.937), 0.897 | 0.817 (0.482 - 1.385), 0.452 |
| Immunologic | 48 | 53 | 19 | 0.954 (0.486 - 1.875), 0.892 | 0.947 (0.576 - 1.559), 0.832 |
| Antinuclear antibody | 105 | 113 | 40 |  |  |

^a^Represents the absolute number of positive patients for F/F, F/f, and f/f genotypes, respectively.

Comparison of genotypes ^b^(F/F + F/f vs f/f genotype) and ^c^(F/F vs Ff + f/f genotype) between patients with and patients without a particular manifestation was performed by ^c^χ^2^ test.

^e^Fisher exact test.

**Table 3S. Prevalence of BsmI (rs1544410) genotypes among SLE patients with different clinical manifestations.**

| **Characteristic** | **Genotype distribution** | | | |  |
| --- | --- | --- | --- | --- | --- |
|  | **B/B**  **(28)^a^** | **b/B**  **(121)^a^** | **b/b**  **(109)^a^** | **Odds ratio (95% CI)^b^, p^c^**  **B/B + b/B vs b/b** | **Odds ratio (95% CI)^d^, p^c^**  **B/B vs b/B + b/b** |
| Malar rash | 5 | 19 | 16 | 1.116 (0.561 - 2.219), 0.754 | 1.211 (0.432 - 3.400), 0.782^e^ |
| Discoid rash | 8 | 34 | 31 | 0.988 (0.571 - 1.709), 0.965 | 1.015 (0.426 - 2.421), 0.973 |
| Phototosensitivity | 16 | 54 | 49 | 1.085 (0.661 - 1.782), 0.747 | 1.644 (0.744 - 3.632), 0.215 |
| Oral or nasopharyngeal | 11 | 47 | 45 | 0.907 (0.548 - 1.500), 0.702 | 0.971 (0.435 - 2.167), 0.942 |
| Arthritis | 4 | 17 | 15 | 1.028 (0.503 - 2.100), 0.939 | 1.031 (0.336 - 3.169), 1.000^e^ |
| Serositis | 3 | 13 | 12 | 0.972 (0.440 - 2.149), 0.945 | 0.984 (0.277 - 3.496), 1.000^e^ |
| Renal | 13 | 61 | 57 | 0.900 (0.549 - 1.476), 0.677 | 0.823 (0.375 - 1.806), 0.626 |
| Neurologic | 5 | 25 | 24 | 0.893 (0.488 - 1.635), 0.713 | 0.803 (0.290 - 2.222), 0.809^e^ |
| Hematologic | 9 | 42 | 37 | 1.013 (0.601 - 1.706), 0.962 | 0.905 (0.391 - 2.095), 0.816 |
| Immunologic | 13 | 58 | 49 | 1.115 (0.679 - 1.830), 0.668 | 0.996 (0.454 - 2.188), 0.993 |
| antinuclear antibody |  |  |  |  |  |

^a^Represents the absolute number of positive patients for b/b, b/B, and B/B genotypes, respectively.

Comparison of genotypes ^b^(b/B + B/B vs b/b genotype) and ^d^(B/B vs b/B + b/b) between patients with and patients without a particular manifestation was performed by ^c^χ^2^ test.

^e^Fisher exact test,

**Table 4S.** **Prevalence of the ApaI (rs7975232) genotypes among SLE patients with different clinical manifestations.**

| **Characteristic** | **Genotype distribution** | | | |  |
| --- | --- | --- | --- | --- | --- |
|  | **A/A**  **(78)^a^** | **A/a**  **(118)^a^** | **a/a**  **(62)^a^** | **Odds ratio (95% CI)^b^, p^c^**  **A/A + A/a vs a/a** | **Odds ratio (95% CI)^d^, p^c^**  **A/A vs Aa + a/a** |
| Malar rash | 13 | 18 | 9 | 1.106 (0.495 - 2.473), 0.805 | 1.133 (0.550 - 2.335), 0.734 |
| Discoid rash | 23 | 33 | 17 | 1.059 (0.559 - 2.005), 0.861 | 1.087 (0.605 - 1.954), 0.780 |
| Phototosensitivity | 38 | 53 | 28 | 1.052 (0.593 - 1.868), 0.862 | 1.161 (0.682 - 1.977), 0.582 |
| Oral or nasopharyngeal | 32 | 45 | 26 | 0.896 (0.502 - 1.601), 0.710 | 1.068 (0.622 - 1.835), 0.812 |
| Arthritis | 11 | 16 | 9 | 0.941 (0.416 - 2.126), 0.883 | 1.018 (0.474 - 2.188), 0.964 |
| Serositis | 9 | 12 | 7 | 0.943 (0.380 - 2.337), 0.899 | 1.105 (0.476 - 2.565), 0.816 |
| Renal | 39 | 60 | 32 | 0.957 (0.540 - 1.695), 0.880 | 0.957 (0.562 - 1.628), 0.870 |
| Neurologic | 16 | 24 | 14 | 0.879 (0.441 - 1.752), 0.714 | 0.964 (0.500 - 1.858), 0.914 |
| Hematologic | 25 | 42 | 21 | 1.014 (0.555 - 1.854), 0.964 | 0.876 (0.498 - 1.542), 0.646 |
| Immunologic | 36 | 56 | 28 | 1.074 (0.605 - 1.906), 0.807 | 0.980 (0.575 - 1.669), 0.940 |
| Antinuclear antibody | 78 | 118 | 62 |  |  |

^a^Represents the absolute number of positive patients for A/A, A/a and a/a genotypes, respectively.

Comparison of genotypes ^b^(A/A + A/a vs a/a genotype) and ^c^(A/A vs Aa + a/a genotype) between patients with and patients without a particular manifestation was performed by ^c^χ^2^ test.

**Table 5S. Prevalence of TaqI (rs731236) genotypes among SLE patients with different clinical manifestations.**

| **Characteristic** | **Genotype distribution** | | | |  |
| --- | --- | --- | --- | --- | --- |
|  | **T/T**  **(108)^a^** | **t/T**  **(122)^a^** | **t/t**  **(28)^a^** | **Odds ratio (95% CI)^b^, p^c^**  **T/T + T/t vs t/t** | **Odds ratio (95% CI)^d^, p^c^**  **T/T vs T/t + t/t** |
| Malar rash | 16 | 19 | 5 | 0.826 (0.294 - 2.318), 0.716 | 0.913 (0.459 - 1.816), 0.795 |
| Discoid rash | 31 | 34 | 8 | 0.985 (0.413 - 2.348), 0.973 | 1.035 (0.598 - 1.792), 0.902 |
| Phototosensitivity | 49 | 55 | 15 | 0.715 (0.326 - 1.571), 0.402 | 0.949 (0.578 - 1.559), 0.837 |
| Oral or nasopharyngeal | 45 | 47 | 11 | 1.030 (0.462 - 2.300), 0.942 | 1.133 (0.684 - 1.876), 0.627 |
| Arthritis | 15 | 17 | 4 | 0.970 (0.316 - 2.980), 1.000^e^ | 0.991 (0.485 - 2.024), 0.980 |
| Serositis | 12 | 13 | 3 | 1.016 (0.286 - 3.611), 1.000^e^ | 1.047 (0.474 - 2.314), 0.910 |
| Renal | 57 | 62 | 12 | 1.429 (0.647 - 3.156), 0.375 | 1.148 (0.700 - 1.884), 0.585 |
| Neurologic | 24 | 25 | 5 | 1.245 (0.450 - 3.445), 0.672 | 1.143 (0.624 - 2.093), 0.665 |
| Hematologic | 37 | 43 | 8 | 1.333 (0.562 - 3.163), 0.513 | 1.012 (0.600 - 1.704), 0.965 |
| Immunologic | 49 | 58 | 13 | 1.004 (0.457 - 2.205), 0.993 | 0.924 (0.563 - 1.518), 0.755 |
| antinuclear antibody | 108 | 122 | 28 |  |  |

^a^Represents the absolute number of positive patients for t/t, t/T, and T/T genotypes, respectively.

Comparison of genotypes ^b^(T/T + t/T vs t/t genotype) and ^d^(T/T vs t/T + t/t) between patients with and patients without a particular manifestation was performed by ^c^χ^2^ test.

^e^Fisher exact test.
