# Supplementary material for: Vitamin D receptor gene BsmI, FokI, ApaI and TaqI polymorphisms and the risk of systemic lupus erythematosus
Source: Mol Biol Rep. 2012 Oct 14;40(2):803–10. doi: 10.1007/s11033-012-2118-6 (PMC3538008; doi:10.1007/s11033-012-2118-6)
Supplement: Supplementary file 2 — Supplementary material 2 (DOC 38 kb) [file 11033_2012_2118_MOESM2_ESM.doc]

**Table 1S. RFLP conditions for the identification of polymorphisms genotyped in the *VDR* gene.**

| **rs no.** | **Alleles** | **Primers for PCR amplification  (5’ – 3’)** | **Annealing temp. (°C)** | **PCR product length (bp)** | **Restriction enzyme** | **Restriction fragment length (bp)** |
| --- | --- | --- | --- | --- | --- | --- |
| rs2228570 | C/T (F/f) | F: GCACTGACTCTGGCTCTGAC | 72.5°C | 341 | FokI | C (F) = 341 |
|  |  | R: ACCCTCCTGCTCCTGTGGCT |  |  |  | T (f) = 282+59 |
| rs1544410 | A/G (B/b) | F: GGAGACACAGATAAGGAAATAC | 60°C | 248 | FspI | A (B) = 248 |
|  |  | R: CCGCAAGAAACCTCAAATAACA |  |  |  | G (b) = 175+73 |
| rs7975232 | A/C (A/a) | F: TGGGCACGGGGATAGAGAAG | 60°C | 177 | ApaI | A (A) = 177 |
|  |  | R: ACGGAGAAGTCACTGGAGGG |  |  |  | C (a) = 131+46 |
| rs731236 | T/C (T/t) | F: TCCTGTGCCTTCTTCTCTATC | 60°C | 172 | TaqI | T (T) = 172 |
|  |  | R: CTAGCTTCTGGATCATCTTGG |  |  |  | C (t) = 107+65 |
